# The Relationship Between the Mathematics Anxiety and Mathematics Achievement of Middle School Students: The Moderating Effect of Working Memory

**DOI:** 10.3390/bs15111566

**Published:** 2025-11-17

**Authors:** Hongye Ma, Changan Sun

**Affiliations:** School of Education, Suzhou University of Science and Technology, Suzhou 215009, China

**Keywords:** mathematics anxiety, mathematics achievement, moderating role, working memory, visual working memory

## Abstract

To investigate the moderating role of working memory subcomponents in the relationship between mathematics anxiety and mathematics achievement among middle school students, this study selected 92 seventh-grade students (45 boys, 47 girls) from a middle school in Suzhou City. The Mathematics Anxiety Scale was used to assess mathematics anxiety levels, while the rotation span task, operation-letter span task, and Stroop task were employed to measure visual working memory, verbal working memory, and central executive system function, respectively. Midterm mathematics exam scores served as the indicator of mathematics achievement. Data were analyzed using correlation analysis and hierarchical regression analysis. The results showed that: (1) Mathematics anxiety was significantly negatively correlated with mathematics achievement (r = −0.61, *p* < 0.01) and had a significant negative predictive effect on mathematics achievement (β = −0.600, *p* < 0.001); (2) Mathematics anxiety was significantly negatively correlated with verbal working memory (r = −0.84, *p* < 0.01), visual working memory (r = −0.68, *p* < 0.01), and the central executive system (r = −0.49, *p* < 0.01), and it had a significant negative predictive effect on all three; (3) Verbal working memory had a significant positive predictive effect on mathematics achievement (β = 0.481, *p* < 0.01); (4) Moderating effect analysis indicated that visual working memory played a significant negative moderating role in the relationship between mathematics anxiety and mathematics achievement (β = −0.226, *p* = 0.017), whereas the moderating effects of verbal working memory and the central executive system were not significant. The research demonstrates that working memory subcomponents play specific roles in the pathway through which mathematics anxiety affects achievement. The resource-dependent nature of visual working memory may exacerbate competition for cognitive resources under anxious conditions, providing empirical evidence for interventions targeting individuals with high visual working memory capacity who experience mathematics anxiety.

## 1. Introduction

### 1.1. Mathematics Anxiety

As a prevalent negative emotional state among student populations, mathematics anxiety has emerged as a global educational challenge (Levine & Pantoja, 2021). The concept was initially introduced by Dreger and Aiken (1957), who observed that students experienced symptoms such as tension, cognitive disorganization, and accelerated heart rate during mathematical problem-solving, ultimately leading to diminished academic performance. Operationally defined, mathematics anxiety typically refers to the feelings of tension, worry, or fear that individuals experience when confronting situations involving numbers, mathematical concepts, or mathematical testing (Geng & Chen, 2005). This form of emotional distress transcends age and geographical boundaries, adversely affecting students across all educational stages, with its manifestations being particularly pronounced during the middle school years (Findings, 2013).

A large amount of empirical evidence consistently shows that there is a robust negative correlation between mathematics anxiety and academic performance. Hembree’s (1990) meta-analysis points out that this association runs from primary school to college; Fu’s (Fu, 2025) integrated research on the basic education stage in China also draws a similar conclusion, and finds that mathematics anxiety has a moderate negative impact on students’ mathematics performance.

It is worth noting that the two-way relationship between mathematics anxiety and mathematics achievement has been gradually supported by empirical evidence. Zhang’s (Zhang et al., 2007), put forward the hypothesis that academic performance may negatively affect emotional state earlier. Subsequently, Sorvo et al. (2019) used tracking data to verify the predictive effect of early mathematics performance on anxiety level one year later, providing preliminary evidence for “the ‘two-way effect’ model” model. Further studies have shown that this two-way relationship is significantly context-dependent (Carey et al., 2016; Szczygieł et al., 2024). A longitudinal study by Szczygieł et al. (2024) found that the effect of low math scores on predicting higher anxiety mainly appeared in students with lower initial academic level or limited working memory resources, but this relationship was not significant in the overall sample. This means that the mechanism of ‘anxiety caused by poor performance’ is not universal, but is regulated by individual cognitive resources and learning experience.

In contrast, the detrimental effect of anxiety on mathematics performance demonstrates greater consistency across diverse populations (Carey et al., 2016). This stable pattern can be explained through the Processing Efficiency Theory and the working memory model: anxious states consume limited cognitive resources, thereby interfering with attentional control and information updating, which ultimately impairs processing efficiency in mathematical tasks (Ashcraft & Krause, 2007).

In summary, the relationship between mathematics anxiety and mathematics achievement is better characterized by a dynamic and individualized bidirectional model. Within this framework, poor performance may exacerbate anxiety under specific conditions, while anxiety, in turn, further undermines mathematical performance through resource depletion. Together, they form a cyclical system moderated by cognitive resources and ability levels. Building upon the cross-cultural and multi-grade research consensus outlined above, this study anticipates that a significant negative correlation between mathematics anxiety and mathematics performance will likewise be confirmed in the current sample of Chinese middle school students.

### 1.2. The Effect of Working Memory on Mathematics Achievement

To further elucidate the cognitive mechanisms through which mathematics anxiety affects mathematical performance, researchers often refer to the working memory model proposed by A. D. Baddeley et al. (1974). This model conceptualizes working memory as comprising a central executive system responsible for monitoring, planning, and strategy selection, along with two subsidiary subsystems: the visuospatial sketchpad, which temporarily stores visual and spatial information, and the phonological loop, which processes verbal and auditory information. The model was later expanded to include the episodic buffer, responsible for integrating information, thereby refining its theoretical framework and sustaining its strong explanatory power (A. Baddeley, 2020).

The predictive role of working memory in mathematics achievement has been widely established (Carr et al., 2020). However, its distinct components play relatively independent roles in mathematical cognition. First, verbal working memory maintains and processes linguistic information relevant to mathematical tasks via the phonological loop. Its functionality is susceptible to disruption by irrelevant auditory input, a phenomenon known as the “irrelevant speech effect” (Allport, 1980). Research by Seyler et al. (2003) demonstrated this mechanism of resource competition: performance significantly declined when individuals were required to memorize letter sequences while simultaneously performing arithmetic operations, underscoring the importance of this component in coordinating cognitive resources. Second, visual working memory, particularly its capacity for spatial representation, is crucial for mathematical understanding and reasoning (Lyons & Beilock, 2009; van Dijck et al., 2022). A strong link is generally acknowledged between mathematical ability and spatial processing (Hawes & Ansari, 2020), with some evidence suggesting that the predictive power of visual working memory for mathematics performance may even exceed that of other subsystems (Giofrè et al., 2017), a finding particularly relevant when solving novel or complex mathematical problems (Szűcs et al., 2014). Finally, the central executive system acts as a higher-order control center, allocating attentional resources and suppressing irrelevant interference (Eysenck et al., 2007). Finell et al. (2022) further highlighted its sensitivity in anxiety-provoking contexts, demonstrating that working memory tasks demanding high levels of executive control showed a substantially stronger negative correlation with mathematics anxiety than simple storage tasks.

In summary, the various subcomponents of working memory support mathematical cognitive activities through distinct pathways. Consequently, this study anticipates a significant positive association between an individual’s overall working memory capacity and their mathematics achievement.

### 1.3. The Theoretical Explanation of the Influence of Mathematics Anxiety on Mathematics Achievement

Regarding how mathematics anxiety impairs mathematical performance, existing theories, from the perspective of cognitive resource allocation and control, provide a multi-level and mutually reinforcing integrative explanatory framework.

First, from a macro resource perspective, the Processing Efficiency Theory (Eysenck & Calvo, 1992) lays the foundational groundwork, positing that anxiety, as a psychological burden, consumes an individual’s limited cognitive resources, thereby directly impairing the efficiency of cognitive processing. Building upon this, the Cognitive Load Theory (Sweller et al., 1998) further specifies this by explicitly defining mathematics anxiety as a form of extraneous cognitive load. This theory emphasizes that this emotionally induced extraneous load interacts with the intrinsic cognitive load of the mathematical task itself; they combine to compete for and deplete limited working memory resources, ultimately leading to the exhaustion of the core mental resources essential for mathematical problem-solving.

Furthermore, from the perspective of specific cognitive processes, the Attentional Control Theory (Eysenck et al., 2007) offers a profound exposition of the intrinsic mechanism of resource depletion. This theory elucidates that in mathematics-anxious situations, an individual’s attention is diverted from the task-focused “goal-directed system” to the threat- and emotion-processing “stimulus-driven system,” resulting in attentional disengagement from the primary task. This failure in attentional control aligns with the mechanism emphasized by inhibition theory—namely, when an individual’s inhibitory function is compromised, they become less effective at suppressing task-irrelevant thoughts and emotional interference elicited by anxiety (Song & Bai, 2003; Tipper, 1985). In such a state, the impaired inhibitory function makes it difficult to suppress anxiety-derived, task-irrelevant thoughts. These intrusive contents then compete with the current mathematical task information for capacity within the working memory space, thereby compromising the effective allocation and deployment of the psychological resources necessary for mathematical cognition.

In summary, these theories systematically demonstrate that mathematics anxiety triggers a cascade: it begins with a breakdown in attentional control, leads to inhibitory failure, manifests as task-irrelevant thoughts that compete for and consume precious working memory resources, and ultimately disrupts the normal operation of the various subsystems underlying mathematical cognition. Consequently, this study proposes the following hypothesis: level of mathematics anxiety will have a significant negative predictive effect on all subsystems of working memory.

### 1.4. Controversy over the Role of Working Memory

As a system with limited resources for temporary information processing and storage, the role of working memory in the relationship between mathematics anxiety and mathematics achievement has become a central research focus. However, existing findings are characterized by a predominance of evidence for mediating effects alongside significant controversy regarding moderating effects. Furthermore, the conclusions on these moderating effects are contradictory. On one hand, the “buffering effect” perspective posits that working memory capacity can attenuate the negative impact of mathematics anxiety on performance. Ashcraft and Kirk’s (2001) study with adults found that individuals with high working memory capacity could allocate resources more efficiently, managing both the demands of the mathematical task and anxiety-related thoughts concurrently, thus experiencing less interference from anxiety. Miller and Bichsel (2004) further corroborated this, showing that highly math-anxious adults with high working memory outperformed their low working memory counterparts in computation and problem-solving tasks. Similarly, Soltanlou et al. (2019), studying elementary schoolchildren, demonstrated that students with low visuospatial working memory suffered more severe impairment in multiplication learning due to mathematics anxiety, whereas those with high visuospatial working memory effectively buffered this negative effect.

On the other hand, the “exacerbating effect” perspective—often termed the “choking under pressure” phenomenon—suggests that high working memory may instead amplify the detrimental effects of mathematics anxiety. Beilock and Carr (2005) argued that individuals with high working memory tend to rely more heavily on memory-based solution strategies in mathematical tasks. The cognitive interference triggered by mathematics anxiety directly disrupts the execution of these strategies, leading to performance that is worse under pressure than that of individuals with lower working memory. Mattarella-Micke et al. (2011) added that this exacerbating effect is particularly pronounced in complex tasks that heavily depend on working memory. Subsequent research focusing on task specificity (Cuder et al., 2023) further refined this understanding: in mathematical fluency tasks (e.g., rapid calculation), visuospatial working memory significantly moderated the mathematics anxiety-performance relationship, with high visuospatial working memory individuals showing greater negative impact from anxiety. In contrast, for mathematical reasoning tasks, visuospatial working memory directly and positively predicted performance, but no moderating effect was observed.

These conflicting findings highlight a fundamental limitation in current research: working memory is not a unitary construct, and its different subcomponents may exert distinct, even antagonistic, moderating influences. Although existing studies have roughly sketched the associative framework of “mathematics anxiety—working memory—mathematics achievement,” three critical research gaps remain, necessitating further exploration: The direction and mechanism of the moderating effects are unclear. The contradictory conclusions regarding whether working memory buffers or exacerbates the impact of anxiety may stem from a failure to systematically compare the specific roles of different working memory subcomponents within the same study. Consequently, the complete picture of their moderating patterns remains obscured. There is a notable scarcity of research specifically investigating the moderating mechanisms of working memory subcomponents in the relationship between mathematics anxiety and achievement among Chinese middle school students. This specific developmental and cultural group is understudied, resulting in a lack of precise empirical evidence to inform targeted educational interventions. Therefore, to resolve the aforementioned controversies and address the deficiencies in existing research, this study will systematically examine the moderating role of working memory subcomponents in the relationship between mathematics anxiety and mathematics achievement among middle school students. It aims to clarify the functional differentiation of these subcomponents, provide a more refined theoretical explanation for the cognitive mechanisms of mathematics anxiety, and offer empirical evidence to guide future intervention practices.

## 2. Research Design

### 2.1. Research Hypotheses

Based on the existing theoretical and empirical research, this study integrates the following core viewpoints: there is a stable negative correlation between mathematics anxiety and academic performance, and its mechanism can be explained by the working memory system. Working memory includes three subsystems, central executive system, verbal working memory and visual working memory, which are responsible for cognitive control, verbal information maintenance and spatial representation, respectively.

The theoretical explanation mainly forms two paths: the perspective of cognitive resources emphasizes that anxiety will compete for limited psychological resources and reduce processing efficiency; from the perspective of executive control, it is believed that anxiety will weaken the ability of attention regulation, resulting in irrelevant information interfering with working memory. It is worth noting that there is a theoretical difference between ‘buffering effect’ and ‘choking under pressure’ in the regulation of working memory, which suggests that the regulation effect may be task-specific and the functions of each subsystem are different. Because of the close relationship between visual working memory and mathematical thinking, it is expected to be particularly prominent in the regulatory mechanism.

Based on the above theoretical framework, this study proposes the following hypotheses to be tested:

**H1.**  *Mathematics anxiety will be significantly negatively correlated with mathematics achievement in middle school students; furthermore, mathematics anxiety will significantly negatively predict mathematics achievement.*

**H2.**  *Mathematics anxiety will be significantly negatively correlated with working memory subcomponents overall in middle school students; furthermore, the level of mathematics anxiety will significantly negatively predict working memory subcomponents.*

**H3.**  *Working memory subcomponents will be significantly positively correlated with mathematics achievement in middle school students; furthermore, working memory subcomponents will significantly positively predict mathematics achievement.*

**H4.**  *Working memory subcomponents will play a moderating role in the relationship between mathematics anxiety and mathematics achievement in middle school students, and different subcomponents will exhibit differential moderating patterns.*

### 2.2. Research Tools

#### 2.2.1. Mathematics Anxiety Scale

The “Mathematics Anxiety Scale” (Xiong, 2008) revised by Xiong (2008) was revised on the basis of MASC according to the cultural background of Chinese middle school students. The revised scale has a total of 21 items. The scale adopts Likert’s five-point scoring method, with very anxiety 5 points and no anxiety 1 point. The higher the score, the higher the level of anxiety. The total score of the scale was 105 points, and the score range of low anxiety was 21–44 points. The range of moderate anxiety was 45~64 points; high anxiety range is higher than 65 points. Examples of scale questions include but are not limited to the following: when you listen to the teacher read a math problem on the blackboard; when you do math homework; and when you are preparing for your final math exam. After SPSS test, the Cronbach’s reliability coefficient is 0.95, and the KMO and Bartlett test coefficient is 0.936. It can reliably measure students’ mathematics anxiety.

#### 2.2.2. Visual Working Memory Task

Visual working memory was assessed using the Rotation Span Task (Wan et al., 2007). The task was programmed and administered using E-Prime 2.0. In each trial, a digit (rotated to 0°, 90°, 180°, or 270°) and an arrow (pointing to a random angle) were presented sequentially for 2000 milliseconds each. Participants were required to type the presented digit. After a sequence of trials concluded, participants had to recall and enter the directions of the arrows from all trials in that sequence, in the correct order.

#### 2.2.3. Verbal Working Memory Task

Verbal working memory was measured using a memory span task based on the operation span task paradigm (Turner & Engle, 1989). Each trial presented an equation-letter pair (e.g., “19 − 7 = 12, Y”) for 4000 milliseconds. Participants were required to judge the veracity of the equation (pressing the F key for correct, J key for incorrect) while simultaneously remembering the letter. After a sequence of trials concluded, participants had to recall and enter all letters from that sequence in the correct order.

#### 2.2.4. Central Executive System Task

According to Baddeley’s working memory model (A. Baddeley, 2020), the central executive system comprises three independent subcomponents: inhibition, updating, and shifting. The inhibitory function is one of its core components. Given its strong representativeness of central executive functioning and high sensitivity, inhibitory control was selected as the assessment focus in this study. The word-color Stroop task (Scarpina & Tagini, 2017) was employed to evaluate the inhibitory function of the central executive system. The task included three conditions: congruent, incongruent, and neutral, with 8 trials for each condition presented in random order. Each trial procedure was as follows: a fixation cross was presented for 500 ms, followed by a colored-word stimulus. Participants were instructed to ignore the word meaning and press the corresponding key to identify the ink color (F key for red, J key for green) within 3000 ms. Inhibitory capacity was measured by the difference in mean reaction times between the incongruent and neutral conditions (MacLeod, 1991).

#### 2.2.5. Mathematics Achievement

Students’ mathematical academic proficiency was measured using their midterm exam scores from the second semester of Grade 7. This score served as the core benchmark, directly reflecting students’ knowledge acquisition and application abilities in mathematics during that specific academic period.

### 2.3. Participants

In this study, 92 students from grade 1 of a junior high school in Suzhou, Jiangsu Province, China, were selected as subjects. The subjects were selected from those who had low, medium, and high mathematics anxiety levels in Phase 1, and met the following conditions: (1) normal intellectual development, no history of mental illness or neurodevelopmental disorders; (2) normal vision or corrected vision; (3) hearing is normal. At the end of the experiment, a small gift was given to the subjects as a thank you. Finally, there were 45 boys, accounting for 48.9%; there were 47 girls, accounting for 51.1 per cent.

### 2.4. Research Procedure

The research procedure consisted of two phases. First, all middle school students were collectively assessed using the Mathematics Anxiety Scale. Based on the scoring criteria, students with low, moderate, and high levels of mathematics anxiety were screened and identified. Subsequently, eligible students were invited to participate in the second phase, which involved working memory tests. This phase was conducted using one-on-one individual testing in a quiet experimental environment. All tasks were administered on 14-inch laptops pre-installed with E-Prime 2.0, and response data were recorded via keyboard input. Each task included both a practice phase and a formal testing phase to ensure participants fully understood the task requirements. All test administrators were uniformly trained postgraduate psychology students to guarantee standardized implementation of the testing procedures.

### 2.5. Statistical Analysis

Data were analyzed using SPSS 25.0. Descriptive statistics, correlation analysis, and tests for common method bias (Harman’s single-factor test) were performed. Hierarchical regression analysis was employed to examine the moderating effects of working memory subcomponents. All continuous predictor variables were mean-centered prior to analysis (Wen et al., 2005).

## 3. Results

### 3.1. Common Method Bias Test

Common method bias was assessed using Harman’s single-factor test. The results showed that four factors had eigenvalues greater than 1. The first factor accounted for 42.89% of the variance, which is below the critical threshold of 50% (Tang & Wen, 2020). This indicates that common method bias was not a serious concern in this study.

### 3.2. Descriptive Statistics and Correlation Analysis

Descriptive statistics and Pearson correlation analyses were conducted to systematically examine the relationships among mathematics anxiety, working memory subcomponents (verbal working memory, visual working memory, and the central executive system), and mathematics achievement. The descriptive statistics for these variables are presented in Table 1. The results indicated considerable individual differences in mathematics achievement among students (SD = 23.95).

The correlation analysis revealed that mathematics anxiety was significantly negatively correlated with mathematics achievement (r = −0.61, *p* < 0.01). It was also significantly negatively correlated with verbal working memory (r = −0.84, *p* < 0.01), visual working memory (r = −0.68, *p* < 0.01), and the central executive system (r = −0.49, *p* < 0.01). Conversely, mathematics achievement was significantly positively correlated with verbal working memory (r = 0.66, *p* < 0.01), visual working memory (r = 0.54, *p* < 0.01), and the central executive system (r = 0.33, *p* < 0.01). Furthermore, the three working memory subcomponents were all significantly positively correlated with each other.

These findings suggest that higher levels of mathematics anxiety are associated with lower mathematics achievement and poorer working memory performance. In contrast, mathematics achievement improves with enhanced working memory capacity, showing a particularly strong association with verbal working memory.

### 3.3. Regression Analysis of Mathematics Anxiety on Working Memory Subcomponents

To examine the predictive effect of mathematics anxiety on working memory subcomponents, hierarchical regression analyses were conducted controlling for gender. Verbal working memory, visual working memory, and the central executive system served as the dependent variables in separate analyses.

The results (see Table 2) indicated that mathematics anxiety had a significant negative predictive effect on all three working memory subcomponents. Specifically, mathematics anxiety significantly negatively predicted verbal working memory (β = −0.835, t = −14.695, *p* < 0.001), accounting for 71.4% of its variance. It also significantly negatively predicted visual working memory (β = −0.683, t = −8.814, *p* < 0.001), accounting for 46.9% of its variance. Furthermore, mathematics anxiety significantly negatively predicted central executive system performance (β = −0.492, t = −5.323, *p* < 0.001), accounting for 24.4% of its variance.

These results demonstrate that higher levels of mathematics anxiety are associated with poorer performance across all measured working memory components. The negative impact of mathematics anxiety was strongest on verbal working memory, followed by visual working memory, and relatively weaker on the central executive system.

### 3.4. Regression Analysis of Mathematics Anxiety and Working Memory on Mathematics Achievement

To systematically examine the predictive effects of mathematics anxiety and working memory subcomponents on mathematics achievement, a hierarchical regression analysis was conducted, controlling for gender. Predictor variables were entered sequentially in the following order: the control variable (gender) in Model 1, mathematics anxiety in Model 2, and the working memory subcomponents in Model 3.

The results (see Table 3) showed that in Model 1, which included only the control variable, gender was not a significant predictor of mathematics achievement. After introducing mathematics anxiety in Model 2, it demonstrated a significant negative predictive effect on mathematics achievement (β = −0.600, t = −7.126, *p* < 0.01). In the final model (Model 3), which included both mathematics anxiety and the working memory subcomponents, only verbal working memory emerged as a significant positive predictor of mathematics achievement (β = 0.481, t = 3.199, *p* < 0.01). The direct predictive effect of mathematics anxiety was no longer significant in this final model.

### 3.5. Moderating Role of Working Memory Subcomponents in the Relationship Between Mathematics Anxiety and Mathematics Achievement

Hierarchical regression analysis was performed using SPSS 25.0 to examine the moderating effects of working memory subcomponents on the relationship between mathematics anxiety and mathematics achievement. All variables were first mean-centered (Wen et al., 2005), and interaction terms were computed before conducting the hierarchical regression. The analysis proceeded in four steps: Step 1 included the control variables, Step 2 added the independent variable, Step 3 introduced the moderator variable, and Step 4 included the interaction term between the independent variable and the moderator.

#### 3.5.1. Moderating Role of Visual Working Memory

To systematically examine the moderating role of visual working memory in the relationship between mathematics anxiety and mathematics achievement, a hierarchical regression analysis was conducted, controlling for gender, verbal working memory, and the central executive system. The results revealed (See Table 4) that in Model 1, which included only the control variables, verbal working memory significantly positively predicted mathematics achievement (β = 0.619, t = 7.184, *p* < 0.001). When mathematics anxiety (Model 2) and visual working memory (Model 3) were added, neither showed a significant main effect (*p* > 0.05). However, upon including the interaction term between mathematics anxiety and visual working memory (Model 4), the interaction effect was significant (β = −0.226, t = −2.425, *p* = 0.017), indicating that visual working memory plays a significant moderating role in the relationship between mathematics anxiety and mathematics achievement.

**Figure 1:**
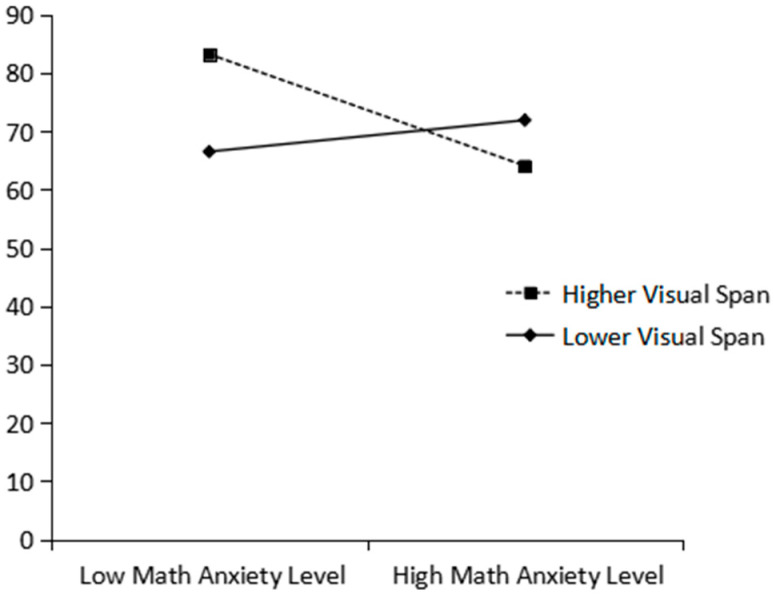
Predictive Effect of the Interaction Between Mathematics Anxiety and Visual Working Memory Capacity on Mathematics Achievement.

To further illustrate the moderating effect of visual working memory capacity, participants were divided into high and low groups based on mean-centered visual working memory scores (scores ±1 SD from the mean), following the procedure by Fang et al. (2015). Simple slope tests (see Figure 1) showed that when visual working memory was low (mean −1 SD), mathematics anxiety did not significantly predict mathematics achievement (simple slope = 0.159, SE = 0.249, t(85) = 0.641, *p* = 0.523). In contrast, when visual working memory was high (mean +1 SD, approximately 1.65), mathematics anxiety showed a marginally significant negative prediction on mathematics achievement (simple slope = −0.566, SE = 0.306, t(85) = −1.851, *p* = 0.068). This pattern of results suggests that higher visual working memory capacity may strengthen the negative association between mathematics anxiety and mathematics achievement. Specifically, a clear negative trend of mathematics anxiety on achievement was only evident when individuals possessed relatively abundant visual working memory resources.

#### 3.5.2. Moderating Role of Verbal Working Memory

Hierarchical regression results indicated (See Table 5) that in Model 1, which included only the control variables, visual working memory significantly positively predicted mathematics achievement (β = 0.476, t = 4.939, *p* < 0.001). When mathematics anxiety was added in Model 2, it demonstrated a significant negative predictive effect on mathematics achievement (β = −0.439, t = −3.631, *p* < 0.001). After introducing verbal working memory in Model 3, it showed a significant positive predictive effect on mathematics achievement (β = 0.481, t = 3.199, *p* = 0.002), while the predictive effect of mathematics anxiety became non-significant (β = −0.053, t = −0.319, *p* = 0.750). Finally, when the interaction term between mathematics anxiety and verbal working memory was included (β = 0.145, t = 1.445, *p* = 0.152), the interaction effect was not statistically significant. This indicates that verbal working memory does not play a significant moderating role in the relationship between mathematics anxiety and mathematics achievement.

#### 3.5.3. Moderating Role of the Central Executive System

The results examining the central executive system as a moderator revealed (See Table 6) that in Model 1, containing only the control variables, both verbal working memory (β = 0.528, t = 5.198, *p* < 0.001) and visual working memory (β = 0.202, t = 2.002, *p* < 0.05) significantly positively predicted mathematics achievement. When the independent variable, mathematics anxiety, was added in Model 2, its predictive effect was not significant (β = −0.086, t = −0.545, *p* > 0.05). Introducing the moderator, the central executive system, in Model 3 also yielded a non-significant main effect (β = 0.056, t = 0.606, *p* > 0.05). Finally, including the interaction term between mathematics anxiety and the central executive system in Model 4 showed a non-significant interaction effect (β = 0.006, t = 0.059, *p* > 0.05). Furthermore, the increase in R^2^ from Model 1 to Model 4 was minimal (from 0.460 to 0.464), indicating that adding the new variables did not substantially improve the model’s explanatory power. Therefore, the central executive system does not serve as a significant moderator in the relationship between mathematics anxiety and mathematics achievement.

## 4. Discussion

### 4.1. Relationship Between Mathematics Anxiety, Mathematics Achievement, and Working Memory Subcomponents

The test of the series of hypotheses in this study shows differentiated results. Assuming that H1 is fully supported, the data show that there is a stable negative correlation between mathematics anxiety and academic performance, and the former has a significant negative predictive effect on the latter. The hypothesis H2 was also verified, and mathematical anxiety showed a significant negative predictive effect on each working memory subsystem. However, assuming that H3 is only partially supported—although correlation analysis showed that all working memory components were positively correlated with math scores, after controlling for other variables, only verbal working memory still maintained independent predictive ability, and the direct predictive effect of visual working memory and central executive system did not reach a significant level.

This study, through Pearson correlation analysis, revealed the association patterns among mathematics anxiety, mathematics achievement, and working memory subcomponents in middle school students. The significant negative correlation between mathematics anxiety and mathematics achievement not only validates the classic phenomenon of “mathematics anxiety accompanying performance decline” (Dreger & Aiken, 1957) but also points to the complex cognitive mechanisms underlying it. Current perspectives suggest that mathematics anxiety consumes and impairs the working memory resources essential for mathematical performance (Ramirez et al., 2018) and may reduce the efficiency of processing verbal and numerical information by creating an attentional bottleneck (Li et al., 2023). From the perspective of cognitive resource allocation, mathematics anxiety, as an emotional load, persistently triggers task-irrelevant worrisome thoughts, thereby occupying and depleting finite cognitive resources. This leads to a significant reduction in the effective mental resources available for mathematical problem-solving (Ashcraft & Kirk, 2001).

Notably, the impact of mathematics anxiety on cognitive function might extend beyond mere “resource consumption” to include a more direct “resource blockade” mechanism. The EEG study by Liu et al. (2020) indicated that individuals with high mathematics anxiety exhibit insensitivity to numerical information in the parietal-occipital P2p component and δ-band neural oscillations during basic numerical processing, suggesting inhibited functionality of the prefrontal-parietal pathway. This implies that mathematics anxiety might block the effective operation of neural pathways from the early stages of information processing. Integrating these findings with our results, mathematics anxiety might initially impair the storage efficiency of verbal working memory via the aforementioned neural mechanism. Subsequently, as individuals generate more anxious thoughts in response to their declining performance, they further consume the already compromised cognitive resources. This creates a vicious cycle where “blockade” and “consumption” pathways act synergistically, jointly exacerbating the decline in mathematics achievement.

Furthermore, hierarchical regression analysis revealed that after controlling for the working memory subcomponents, the direct predictive effect of mathematics anxiety on mathematics achievement was no longer significant, whereas verbal working memory emerged as a significant predictor. This pattern of results suggests that verbal working memory may serve a crucial mediating function between mathematics anxiety and mathematics achievement. Mathematics anxiety appears to indirectly impair mathematical performance primarily by consuming verbal working memory resources (Ashcraft & Krause, 2007). This finding resonates with the conclusions of Živković et al. (2023) regarding the mediating role of working memory, despite differences in the specific subcomponents involved (visuospatial working memory in their study). Collectively, these results support the theoretical view of working memory as a core cognitive pathway through which mathematics anxiety affects achievement. Differences at the subcomponent level might stem from variations in study populations or task types, yet they consistently highlight the pivotal role of working memory in the mechanism of mathematics anxiety.

This study also found that visuospatial working memory plays a moderating role in the relationship between mathematics anxiety and mathematics achievement, manifesting as a negative moderating effect. Paradoxically, higher visuospatial working memory capacity strengthened the negative impact of mathematics anxiety on achievement. This result aligns with the “choking under pressure” theory (Beilock & Carr, 2005) and the findings of Cuder et al. (2023) regarding task dependency, indicating that the moderating effect of visual working memory is particularly pronounced in tasks with lower visuospatial demands. For seventh-grade students, who are in a transitional period of mathematical learning, high visuospatial working memory might lead to counterproductive effects under anxiety due to the non-essential investment of cognitive resources, reflecting a mechanism where cognitive resources are inefficiently consumed under the interference of anxiety (Caviola et al., 2017).

### 4.2. The Moderating Role of Working Memory Subcomponents in the Relationship Between Mathematics Anxiety and Mathematics Performance

Regarding the central hypothesis (H4) on the moderating role of working memory, this study reveals a clear functional dissociation among its subcomponents. Hypothesis H4 was partially supported: visuospatial working memory demonstrated a significant negative moderating effect, indicating that higher levels of this capacity were associated with a stronger detrimental impact of mathematics anxiety on performance. In contrast, neither verbal working memory nor the central executive system exhibited significant moderating effects. These findings underscore the high degree of component specificity in the regulatory function of working memory, suggesting that not all subsystems are involved in moderating this cognitive-emotional process.

Of note, visuospatial working memory did not buffer the negative impact of mathematics anxiety as expected; instead, it exacerbated the detrimental effect of anxiety on mathematics performance. This finding challenges the traditional view that working memory generally exerts a protective effect (e.g., Foley et al., 2017) and lends support to the explanatory framework of the “choking under pressure” theory (Peng & Ma, 2025). From a cognitive mechanism perspective, individuals with high visuospatial working memory typically rely on efficient spatial representation and strategic processing, a mode that itself consumes considerable visuospatial resources. When mathematics anxiety is activated, the worrisome thoughts and emotional responses it elicits function as an extraneous cognitive load, further competing for limited cognitive resources, thereby creating a resource competition bottleneck in the predominant neural circuits. This competition leads to reduced precision in spatial encoding and disruption of the strategic processing flow, ultimately amplifying the negative impact of anxiety, manifesting as a typical “performance collapse” under high cognitive load.

In contrast to visuospatial working memory, the moderating effect of verbal working memory in this study was not significant. This suggests that verbal working memory may primarily play a mediating role. Furthermore, its main effect on mathematics performance was substantially stronger than its moderating effect. This indicates that verbal working memory influences performance mainly through direct resource support, and the interference from mathematics anxiety tends to manifest as a general consumption of cognitive resources rather than a specific interaction.

No significant moderating effect was found for the central executive system in this study. It is important to note that we only measured inhibitory ability and did not assess the other two proposed functions of the central executive system. Future research could investigate the roles of these additional central executive functions in the relationship between mathematics anxiety and mathematics performance.

### 4.3. Limitations and Future Directions

This study has several limitations. First, the sample was drawn from junior high school students in a single geographic region, which limits the generalizability of the findings. Second, the potential influence of grade-level differences on the moderating effects was not examined. Third, the measurement of the central executive system using the Stroop task did not reveal significant associations; future studies should employ more sensitive paradigms, such as the N-back task, for a more in-depth investigation. It is recommended that future research expand sample representativeness, incorporate longitudinal tracking across multiple educational stages, and develop intervention training targeting specific working memory subcomponents to alleviate mathematics performance impairment in highly anxious individuals.

Furthermore, when interpreting the findings of this study and considering their educational applications, it is necessary to recognize the inherent complexity of the cognitive load theory framework upon which it relies. For instance, Schnotz and Kürschner (2007) pointed out that systematically reducing extraneous cognitive load does not always lead to improved learning outcomes in authentic learning environments. This reminds us that transitioning from the experimental context of this study to dynamic and complex real-world classrooms necessitates consideration of additional factors regarding the impact of mathematics anxiety and its intervention. Therefore, a cautious approach is warranted when translating explanations based on cognitive resource allocation into educational practice.

## 5. Conclusions

This study systematically examined the relationships between mathematics anxiety, working memory subcomponents, and mathematics performance among junior high school students. The main conclusions are as follows:

(1) Mathematics anxiety was significantly negatively correlated with mathematics performance in adolescents. It significantly negatively predicted mathematics performance when working memory was not controlled for; however, this direct predictive effect became non-significant after controlling for working memory.
(2) Mathematics anxiety was significantly negatively correlated with verbal working memory, visuospatial working memory, and the central executive system, and it significantly negatively predicted all three subcomponents. The negative impact was strongest on verbal working memory.
(3) Verbal working memory, visuospatial working memory, and the central executive system were all significantly positively correlated with mathematics performance. However, only verbal working memory significantly positively predicted mathematics performance.
(4) Only visuospatial working memory played a significant negative moderating role in the relationship between mathematics anxiety and mathematics performance, exacerbating the negative effect of anxiety on performance. No significant moderating effects were found for verbal working memory or the central executive system.

This research reveals the specificity of working memory subcomponents within the mechanism of mathematics anxiety, provides new empirical evidence for understanding the “choking under pressure” phenomenon, and suggests that future educational interventions should adopt differentiated strategies tailored to anxious students with varying working memory profiles.

## Figures and Tables

**Table 1:** Descriptive Statistics and Correlations among Variables.

	M	SD	Mathematics Anxiety	Mathematics Achievement	Verbal Working Memory	Visual Working Memory	Central Executive System
Mathematics Anxiety	54.45	16.95	1				
Mathematics Achievement	72.25	23.95	−0.61 **	1			
Verbal Working Memory	4.91	1.70	−0.84 **	0.66 **	1		
Visual Working Memory	5.44	1.65	−0.68 **	0.54 **	0.63 **	1	
Central Executive System	50.16	25.05	−0.49 **	0.33 **	0.36 **	0.39 **	1

Note. ** *p* < 0.01 (two-tailed).

**Table 2:** Regression Analysis of Mathematics Anxiety on Working Memory Subcomponents.

Dependent Variable	Predictor	β	t	p	R^2^	Adjusted R^2^	F
Verbal Working Memory	Mathematics Anxiety	−0.835	−14.695	<0.001	0.714	0.708	F(2,89) = 111.31 ***
Visual Working Memory	Mathematics Anxiety	−0.683	−8.814	<0.001	0.469	0.457	F(2,89) = 39.23 ***
Central Executive System	Mathematics Anxiety	−0.492	−5.323	<0.001	0.244	0.227	F(2,89) = 14.39 ***

Note. *** *p* < 0.001.

**Table 3:** Hierarchical Regression Analysis Predicting Mathematics Achievement.

Predictor	Model 1		Model 2		Model 3	
	β	t	β	t	β	t
**Step 1: Control Var.**						
Gender	−0.123	−1.176	−0.079	−0.937	−0.033	−0.415
**Step 2: Main Effect**						
Mathematics Anxiety			−0.600	−7.126	−0.053	−0.319
**Step 3: WM Subcomp.**						
Verbal Working Memory					0.481	3.199
Central Executive System					0.173	1.574
Visual Working Memory					0.056	0.606
R^2^	0.015		0.373		0.464	
Adjusted R^2^	0.004		0.359		0.433	
F	F = 1.383		F = 26.466		F = 14.871	

**Table 4:** Hierarchical Regression Analysis Testing the Moderating Effect of Visual Working Memory.

Predictor	Model 1		Model 2		Model 3		Model 4	
	β	t	β	t	β	t	β	t
**Step 1: Control Variables**								
Gender	−0.026	−0.325	−0.033	−0.411	−0.033	−0.415	−0.047	−0.600
Verbal Working Memory	0.619	7.184	0.514	3.426	0.481	3.199	0.336	2.129
Central Executive System	0.100	1.172	0.070	0.764	0.056	0.606	0.093	1.029
**Step 2: Independent Variable**								
Mathematics Anxiety			−0.136	−0.854	−0.053	−0.319	−0.144	−0.864
**Step 3: Moderator**								
Visual Working Memory					0.173	1.574	0.093	0.830
**Step 4: Interaction**								
Math Anxiety × Visual Working Mem							−0.226	−2.425
R^2^	0.444		0.448		0.464		0.498	
Adjusted R^2^	0.425		0.423		0.433		0.463	
F	F = 23.389		F = 17.670		F = 14.871		F = 14.076	

**Table 5:** Moderating Role of Verbal Working Memory in the Mathematics Anxiety-Achievement Relationship.

Predictor	Model 1		Model 2		Model 3		Model 4	
	β	t	β	t	β	t	β	t
**Step 1: Control Variables**								
Gender	−0.082	−0.923	−0.074	−0.892	−0.033	−0.415	−0.043	−0.540
Visual Working Memory	0.476	4.939	0.222	1.944	0.173	1.574	0.295	2.137
Central Executive System	0.134	1.395	0.018	0.188	0.056	0.606	0.042	0.462
**Step 2: Independent Variable**								
Mathematics Anxiety			−0.439	−3.631	−0.053	−0.319	0.037	0.211
**Step 3: Moderator**								
Verbal Working Memory					0.481	3.199	0.481	3.223
**Step 4: Interaction**								
Math Anxiety × Verbal Working Mem							0.145	1.445
R^2^	0.309		0.400		0.464		0.477	
Adjusted R^2^	0.285		0.372		0.433		0.440	
F	F = 13.11		F = 14.492		F = 14.841		F = 12.898	

**Table 6:** Moderating Role of the Central Executive System in the Mathematics Anxiety-Achievement Relationship.

Predictor	Model 1		Model 2		Model 3		Model 4	
	β	t	β	t	β	t	β	t
**Step 1: Control Variables**								
Gender	−0.032	−0.401	−0.036	−0.446	−0.033	−0.415	−0.033	−0.402
Visual Working Memory	0.202	2.002	0.180	1.650	0.173	1.574	0.175	1.526
Verbal Working Memory	0.528	5.198	0.469	3.159	0.481	3.199	0.482	3.172
**Step 2: Independent Variable**								
Mathematics Anxiety			−0.086	−0.545	−0.053	−0.319	−0.052	−0.309
**Step 3: Moderator**								
Central Executive System					0.056	0.606	0.052	0.457
**Step 4: Interaction**								
Math Anxiety × Central Executive Sys							0.006	0.059
R^2^	0.460		0.461		0.464		0.464	
Adjusted R^2^	0.441		0.437		0.433		0.426	
F	F = 24.944		F = 18.633		F = 14.871		F = 12.250	

## Data Availability

The datasets generated and analyzed during the current study are available from the corresponding author on reasonable request. The experimental scripts used in this study were created with E-Prime 2.0. Owing to the license agreement of E-Prime 2.0, these scripts cannot be publicly redistributed. However, the corresponding author will provide the scripts upon reasonable request for the sole purpose of replicating the experimental procedure.
